# Ecological Niche Modeling of *Hoplias malabaricus* (Characiformes, Erythrinidae) Under Climate Change Scenarios

**DOI:** 10.1002/ece3.72361

**Published:** 2025-10-30

**Authors:** Karen L. Auzier Guimarães, Sarah J. do Nascimento Andrade, Luís R. Ribeiro Rodrigues

**Affiliations:** ^1^ Programa de Pós‐Graduação em Biodiversidade e Biotecnologia (REDE BIONORTE), Instituto de Saúde Coletiva (ISCO) Universidade Federal do Oeste do Pará (UFOPA) Santarém Pará Brazil; ^2^ Laboratório de Genética e Biodiversidade (LGBio), Instituto de Ciências da Educação (ICED) Universidade Federal do Oeste do Pará (UFOPA) Santarém Pará Brazil; ^3^ Centro Avançado de Pesquisa‐Ação da Conservação e Recuperação Ecossistêmica da Amazônia (CAPACREAM) Universidade Federal do Amapá (UNIFAP) Macapá AP Brazil; ^4^ Programa de Pós‐Graduação em Biodiversidade (PPGBEES), Instituto de Ciências e Tecnologia das Águas (ICTA) Universidade Federal do Oeste do Pará (UFOPA) Santarém Pará Brazil

**Keywords:** freshwater fish, habitat suitability, MaxEnt, South America, species distribution

## Abstract

Freshwater fish are highly vulnerable to climate change because they are restricted to inland waters and cannot readily disperse across geographical barriers, making them particularly exposed to catastrophic habitat losses. Understanding how environmental changes may affect the distribution of widespread species is critical for anticipating biodiversity responses and informing conservation efforts. In this study, we employed ecological niche models (MaxEnt) to assess the current and future potential distribution of *Hoplias malabaricus* , a generalist freshwater predator of ecological and fisheries importance in South America. We focus on the Amazon, Tocantins–Araguaia, Guiana Shield, Brazilian Atlantic Coast, and Marajó Island basins, which encompass the current distribution of the species. We used bioclimatic variables derived from the global WorldClim v2.1 dataset under present‐day conditions and two future climate scenarios (SSP1–2.6, a low‐emission pathway, and SSP5–8.5, a high‐emission pathway) to project habitat suitability. The models demonstrated excellent predictive performance (AUC > 0.9), identifying temperature seasonality (BIO4) and elevation as the most influential variables across all scenarios. Results revealed contrasting trends among hydrographic regions. The species showed increasing association with more seasonal environments and highly suitable habitats contracted under the most extreme scenario. Marajó Island exhibited the highest loss of suitable area, highlighting increased isolation risks. Overall, our findings indicate that *H. malabaricus* may persist under climate change through niche shifts and partial range contractions. However, habitat loss, reduced connectivity, and regional genetic isolation may compromise long‐term viability, particularly under high‐emission scenarios.

## Graphical abstract

This study employs ecological niche models (ENMs) to project current and future habitat suitability for a genetically validated lineage of *Hoplias malabaricus* , a widely distributed Neotropical freshwater predator.

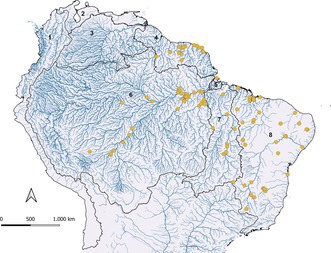

## 1 Introduction

The persistence and integrity of freshwater ecosystems depend directly on climatic and hydrological regimes. Changes in temperature and precipitation patterns can alter flow dynamics, water availability, and habitat structure, making freshwater environments and the organisms that depend on them particularly sensitive to climate change (Morrongiello et al. 2011). Many species may face local or regional extirpation if current trends persist (Wiens 2016). Among aquatic taxa, fish are especially vulnerable due to their strong physiological and ecological dependence on water temperature, dissolved oxygen, and seasonal hydrological cycles (Dahlke et al. 2020; Mariu et al. 2023). Broadly, species can respond to climatic shifts in three ways: by adapting to new conditions, by shifting their distribution to track suitable environments, or by facing population collapse and potential extinction (Olusanya and van Zyll de Jong 2018).

In this context, species distribution models (SDMs) are widely used to assess current and future areas of suitable habitat for species, based on assumptions of ecological niche theory. These models serve as tools for predicting how species may respond to climate change (Kim et al. 2020; Hu et al. 2022) and have been successfully applied in both marine (Wang et al. 2018; Silva et al. 2019) and freshwater ecosystem studies (Schmidt et al. 2020; Aksu 2021). Identifying vulnerable species, ecosystems, and habitats remains essential for guiding conservation efforts and for advancing the understanding of climate change impacts and reducing its effects on biodiversity (Williams et al. 2008).

The Amazon and surrounding ecoregions harbor some of the world's most diverse freshwater fish assemblages (Jézéquel et al. 2020; Cassemiro et al. 2023), yet face increasing pressures from climate change, deforestation, dam construction, and other anthropogenic stressors (Albert et al. 2023). Despite the recognized biodiversity and ecological complexity of these systems, data on species‐level responses to climate change remain limited (Frederico et al. 2021).

*Hoplias malabaricus* (Bloch 1794), commonly known as trahira, is a widely distributed neotropical freshwater fish found across multiple South American hydrographic basins and occupies a broad range of habitats such as lakes, lagoons, small and large rivers (Oyakawa 2003; Cardoso et al. 2018; Guimarães et al. 2022). It is typically associated with structurally complex habitats, such as riparian‐vegetation margins, submerged macrophytes, and woody debris, which provide shelter for its ambush‐predator behavior by attracting small fish seeking refuge, thereby increasing its predation efficiency in these environments (Corrêa et al. 2012). The species exhibits high ecological resilience, tolerating a broad range of hydrological conditions and relatively low dissolved oxygen concentrations (Soares et al. 2006). It often thrives in lentic or slow‐flowing habitats and its feeding and reproductive dynamics are influenced by seasonal flood pulses (De Oliveira et al. 2014). Despite its ecological versatility, recent studies have revealed genetic structuring among populations, with distinct lineages often confined to specific basins (Guimarães et al. 2022). This combination of ecological versatility and regional genetic divergence makes *H. malabaricus* an ideal model for evaluating climate change impacts, while also allowing for more localized assessments of vulnerability and resilience.

In this study, we use SDMs to evaluate the current and future potential distribution of *H. malabaricus* under contrasting climate change scenarios (SSP1–2.6 and SSP5–8.5). The SSP1–2.6 scenario represents an optimistic projection, with low levels of greenhouse gas emissions, while SSP5–8.5 corresponds to a pessimistic projection, characterized by high emissions of these gases (O'Neill et al. 2014). We focus on the Amazon, Tocantins–Araguaia, Guiana Shield, Brazilian Atlantic Coast, and Marajó Island basins, which encompass the current distribution of the species. By analyzing habitat suitability across major hydrographic basins, we aim to identify areas of persistence, contraction, and potential climatic refugia. Specifically, we ask: (1) How will climate change affect the extent and spatial configuration of suitable and highly suitable habitats for this species? (2) Are there regional differences in the species' responses among basins, indicating unequal vulnerabilities? (3) What are the ecological and conservation implications of projected habitat changes?

## 2 Materials and Methods

### 2.1 Description of the Model Species and Occurrence Records

**FIGURE 1:**
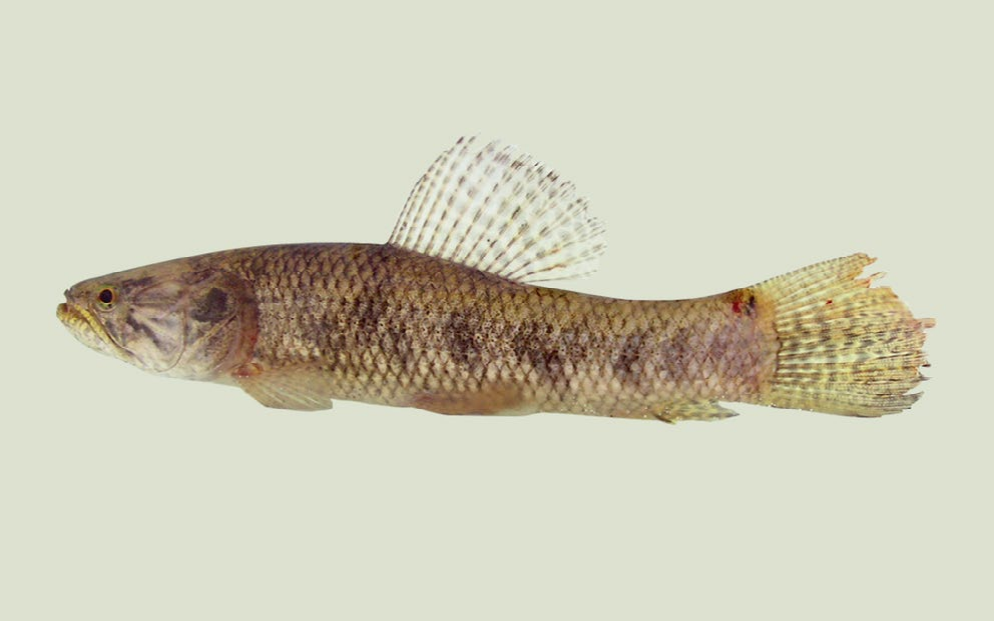
Lateral view of *Hoplias malabaricus* , showing the body shape and color pattern of the species. Photo by Luís Rodrigues.

We modeled the distribution of *H. malabaricus* (Figure 1), focusing on a genetically validated mitochondrial lineage registered in the Barcode of Life Data Systems (BOLD:AFU2064). This taxon is part of a widely distributed species complex that exhibits extensive cryptic speciation, with multiple deeply divergent lineages occurring across South America (Cardoso et al. 2018; Guimarães et al. 2022). Among these, lineage BOLD:AFU2064 includes specimens from the type locality of *H. malabaricus* , justifying its designation as the *sensu stricto* lineage (Cardoso et al. 2018). It also benefits from extensive occurrence records, which enhance the reliability of ecological niche modeling. Given these attributes, we hereafter treat BOLD:AFU2064 as an independent species for modeling purposes.

*Hoplias malabaricus* is a carnivorous, ambush predator with a broad ecological role in freshwater ecosystems. While the *H. malabaricus* complex is often described as ecologically generalist, individual lineages may exhibit more specific environmental requirements and narrower ecological niches. Accordingly, conservation and climate modeling efforts should explicitly account for genetic diversity, as treating all populations under a single taxonomic entity may obscure lineage‐specific vulnerabilities and adaptive responses (Bothwell et al. 2021).

**FIGURE 2:**
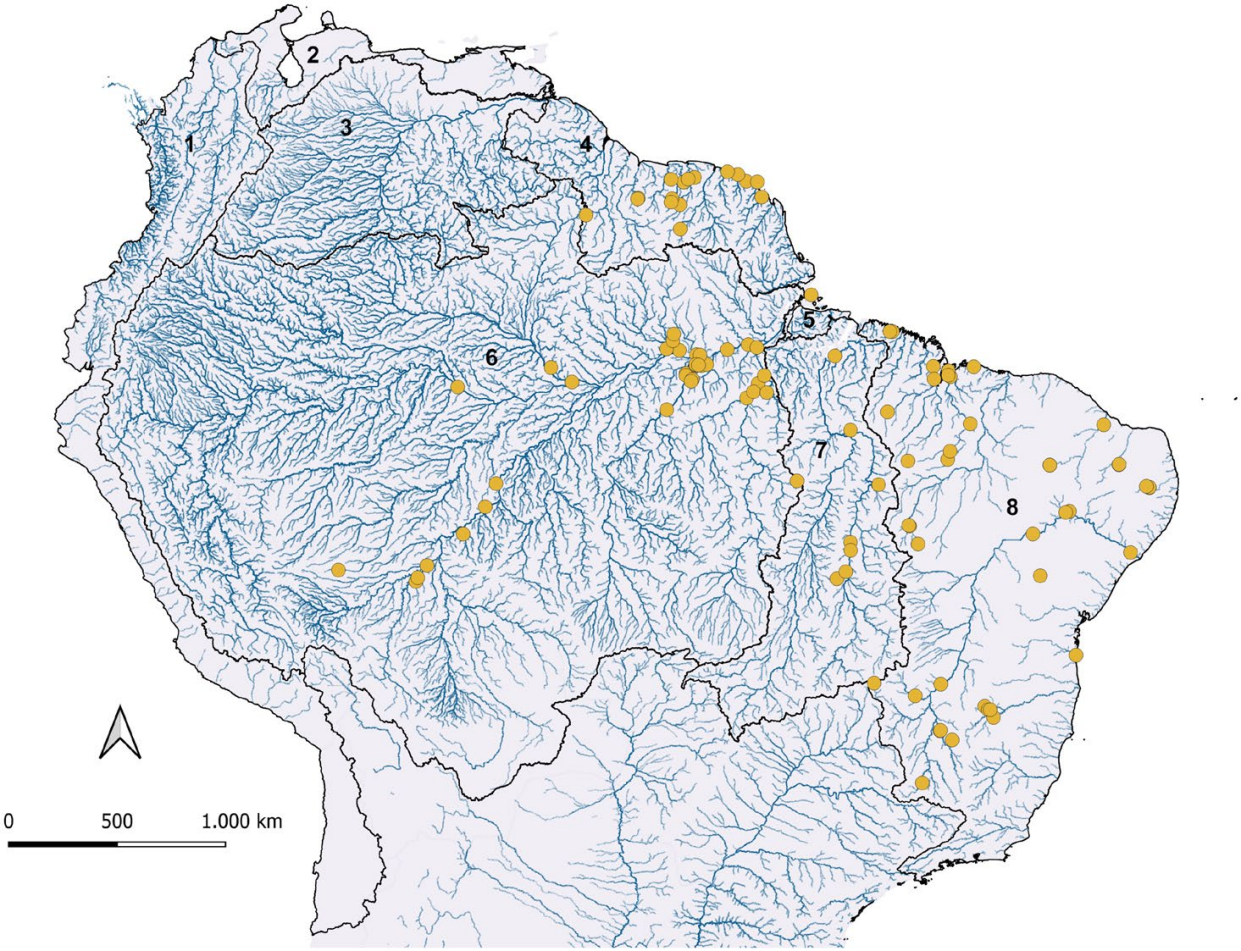
Georeferenced occurrence records of DNA barcoded (COI) species *Hoplias malabaricus* (BOLD:AFU2064). Major hydrographic basins are indicated by numbers: (1) Pacific Coast, (2) Maracaibo–Caribbean, (3) Orinoco, (4) Guiana Shield, (5) Marajó Island, (6) Amazon, (7) Tocantins–Araguaia, and (8) Brazilian Atlantic Coast. Figure created with QGIS version 3.36.3 (Quantum GIS Development Team, www.qgis.org).

Occurrence records used in this study were obtained from previously published datasets (Cardoso et al. 2018, Guimarães et al. 2022) and complemented by our own barcoded specimens, all confirmed through mitochondrial DNA barcoding (COI gene) (Figure 2). This approach minimizes the risk of misidentification and ensures that the ecological niche modeling accurately reflects the environmental preferences of a single evolutionary lineage within the *H. malabaricus* species complex.

We compiled a total of 107 georeferenced occurrence records of *H. malabaricus* (BIN BOLD:AFU2064), distributed across the following major hydrographic basins: Amazon (*n* = 38), Tocantins–Araguaia (*n* = 7), Guiana Shield (*n* = 16), Brazilian Atlantic Coast (*n* = 45), Marajó Island (*n* = 1). Basin delimitations were based on the HydroSHEDS database (Lehner and Grill 2013), which provides globally standardized watershed boundaries.

The Amazon Basin is the largest and most diverse freshwater system in the world, with extensive floodplains and high fish diversity (Reis et al. 2016; Correa et al. 2025). The Tocantins–Araguaia Basin is formed mainly by the Tocantins and Araguaia rivers, originating in the Central Plateau, with distinct elevation gradients and seasonal hydrology across highland, central, and lower reaches (Penereiro et al. 2015; Chamon et al. 2022). The Guiana Shield is an ancient Proterozoic‐Archean formation spanning northeast South America, characterized by tepuis, waterfalls, intact forests, and high endemism (Potapov et al. 2017; De Souza et al. 2020). The Brazilian Atlantic Coast basins comprise several sub‐basins including São Francisco, western Northeast Atlantic, Northeast Atlantic Eastern, Parnaíba, Atlantic East, and Atlantic Southeast. These basins encompass a range of climatic conditions, from semi‐arid regions with prolonged dry periods to humid tropical forests, and display varied topography including lowland floodplains, plateaus, and coastal plains. They host distinct biomes, including Cerrado savannas, Caatinga drylands, and Atlantic Forest remnants, which provide heterogeneous freshwater habitats such as rivers, streams, wetlands, and floodplain lagoons, supporting rich and diverse fish communities (Ramos et al. 2014; Lima et al. 2020; Macêdo et al. 2023; Andrade et al. 2024; Guimarães et al. 2025). Marajó Island, at the mouth of the Amazon River, is a large fluvial island with seasonally flooded plains and estuarine influence, providing a unique habitat for freshwater fishes (Cohen et al. 2008; Montag et al. 2009). This diversity of hydrographic and ecological settings provides a representative spatial framework for modeling the ecological niche of *H. malabaricus* .

### 2.2 Climate Change Scenarios and Data Analysis

To simulate the current and future distribution of *H. malabaricus* , we employed MaxEnt version 3.4.4k, a species distribution modeling software based on the principle of maximum entropy (Phillips et al. 2006). Nineteen bioclimatic variables representing annual trends were obtained from the WorldClim version 2 database at a spatial resolution of 30 arc‐seconds (~1 km^2^) (Fick and Hijmans 2017). In addition, one topographic variable considered to be stable over time, the hydrologically conditioned digital elevation model (DEM), was included, obtained from the HydroSHEDS database (https://www.hydrosheds.org/hydrosheds‐core‐downloads).

To reduce multicollinearity among the bioclimatic predictors and improve model performance, we applied a variance inflation factor (VIF) analysis prior to model calibration. VIF is a diagnostic tool that quantifies the degree of collinearity between each variable and all others in a multiple regression context (O'Brien 2007). Variables with VIF values greater than 10 are commonly considered highly collinear and were excluded from the final model to avoid inflation of variance and overfitting. After excluding highly correlated variables, the final set used for model calibration included ten predictors with acceptable VIF values, ranging from 2.30 to 9.30: Isothermality (BIO3), temperature seasonality (BIO4), max temperature of warmest month (BIO5), mean temperature of wettest quarter (BIO8), precipitation of wettest month (BIO13), precipitation of driest month (BIO14), precipitation seasonality (BIO15), precipitation of warmest quarter (BIO18), precipitation of coldest quarter (BIO19), and hydrographically conditioned elevation model (DEM). This procedure ensures that the retained variables contribute independent information to the model and minimize redundancy. The same set of uncorrelated variables was used for modeling current and future scenarios.

We used 75% of occurrence data for training and 25% for testing the model. The final suitability maps were generated by taking the median output of 10 bootstrap replicates. Future projections of species distribution were carried out using climate data derived from a general circulation model (GCM) included in the coupled model intercomparison project phase 6 (CMIP6). This framework simulates future climatic conditions under different greenhouse gas emission pathways (Eyring et al. 2016; Feng et al. 2020). Two shared socioeconomic pathways (SSPs) were considered: SSP1–2.6 and SSP5–8.5. The SSP1–2.6 represents an optimistic scenario in which human development transitions toward sustainability and lower emissions, while SSP5–8.5 assumes a high‐emission trajectory driven by rapid economic growth and energy consumption (O'Neill et al. 2014). Predictions for future species distributions were generated for the period 2081–2100, using the same spatial resolution of 30 arc‐seconds. All preprocessing of spatial data, including manipulation of raster and vector layers, was performed using QGIS version 3.36.3 (Quantum GIS Development Team, www.qgis.org).

### 2.3 Model Evaluation and Habitat Suitability Interpretation

To evaluate model performance, we analyzed the area under the curve (AUC) values of the receiver operating characteristic (ROC) curves. The AUC quantifies the overall ability of the model to discriminate between suitable and unsuitable habitats across all threshold values. The closer the AUC test value is to 1, the better the model's sensitivity and discriminatory power. According to standard classifications, AUC values are interpreted as follows: failure (0.5–0.6), poor (0.6–0.7), fair (0.7–0.8), good (0.8–0.9), and excellent (0.9–1) (Swets 1988; Phillips et al. 2006).

The importance of environmental variables in shaping the species distribution was assessed based on the percentage contribution and permutation importance of metrics provided by MaxEnt. These metrics indicate how much each variable contributes to model performance and help determine their relative significance in predicting habitat suitability.

Model outputs were interpreted as relative habitat suitability and classified into four thresholds: unsuitable (0–0.25), low suitability (0.25–0.50), suitable (0.50–0.75), and highly suitable (0.75–1). Spatial analyses and calculations of the total area corresponding to each suitability class were performed using QGIS version 3.36.3. Additionally, for each scenario, environmental variable values were extracted from the raster layers for pixels corresponding to the “suitable” (0.50–0.75) and “highly suitable” (0.75–1) habitat classes, to allow ecological interpretation of the species environmental preferences (Çoban et al. 2020; Roy et al. 2021).

To explore spatial variation in environmental suitability across the species' range, we conducted basin‐level analyses of suitable and highly suitable areas under current and future climate scenarios. We aimed to identify differences in habitat quality and extent across major hydrographic basins, potentially reflecting distinct regional responses to climatic drivers. For each basin, values of the environmental variables used in the models were extracted from the raster outputs generated by MaxEnt for two suitability classes: 0.50–0.75 (suitable) and 0.75–1.0 (highly suitable). This extraction was carried out for all three scenarios: current climate, SSP1–2.6, and SSP5–8.5. Additionally, the area (in km^2^) corresponding to these classes was calculated for every basin and scenario. All spatial analyses, including variable extraction and area calculations, were performed using QGIS version 3.36.3 (QGIS Development Team 2024), with the aid of raster calculator and zonal statistics tools.

## 3 Results

### 3.1 Model Performance

The predictive performance of the species distribution models was evaluated using the AUC metric. The results indicated high predictive accuracy across all climatic scenarios. For the present scenario, the average training AUC value across replicate runs was 0.936 (±0.006). Under the SSP1–2.6 future climate scenario, the average AUC increased slightly to 0.937 (±0.012). The highest model performance was observed in the SSP5–8.5 scenario, with an average AUC of 0.949 and a standard deviation of 0.003.

### 3.2 Current and Future Potential Spatial Distributions of *H. malabaricus*

The predicted suitable habitats for *H. malabaricus* under current climate conditions are illustrated in Figure 4A. Known occurrence records were largely congruent with areas predicted as suitable by the model. The species' known distribution, encompassing the Brazilian Atlantic Coast, Guianas Shield, Amazon, Marajó Island, and Tocantins‐Araguaia basins, closely matches the model's outputs. Additionally, the model projected suitable conditions in regions where no confirmed occurrences have been recorded to date including the Orinoco, Pacific Coast, and Maracaibo‐Caribbean basins.

Under the SSP1–2.6 scenario, the most influential environmental variables based on percent contribution were elevation (31.5%), temperature seasonality (BIO4) (16.7%), precipitation seasonality (BIO15) (14.8%), and precipitation of the warmest quarter (BIO18) (10.4%). In terms of permutation importance, the most relevant variables were elevation (29%), precipitation of the coldest quarter (BIO19) (15.2%), BIO18 (11.3%), and BIO4 (10.2%). Although BIO15 showed a lower permutation importance (7.3%), its high percent contribution underscores its role in the model. On the other hand, BIO19, despite a modest percent contribution (8.3%), had a high permutation importance (15.2%), indicating its strong influence on the model predictive performance (Table 1).

**TABLE 1:** Percent contribution (PC) and permutation importance (PI) of environmental variables used in the model for *Hoplias malabaricus* (BOLD:AFU2064).

Variable	Present		SSP1–2.6		SSP5–8.5	
	PC	PI	PC	PI	PC	PI
DEM	**33.4**	**17.5**	**31.5**	**29**	**31.9**	**37.5**
BIO3	5	6.1	5.3	8.3	2.2	4.5
BIO4	**22.8**	**19.6**	**16.7**	**10.2**	**17.7**	**22.5**
BIO5	1.9	4.6	1.9	3.5	1.5	2.9
BIO8	2.7	2.9	3.8	5.2	1.5	2.8
BIO13	2.8	3.5	5.3	5.6	5.6	3.3
BIO14	3.6	9.8	2.2	4.3	3.5	0.9
BIO15	6.1	5.2	**14.8**	7.3	**22.2**	**10.8**
BIO18	**13.3**	**19.8**	**10.4**	**11.3**	9.4	6.5
BIO19	8.4	11	8.3	**15.2**	4.4	8.4

*Note:* Values above 10% are highlighted in bold, indicating greater influence on model performance across scenarios.

Under the SSP5–8.5 scenario, elevation remained the most influential environmental variable, with a percent contribution of 31.9% and a high permutation importance of 37.5%. Temperature seasonality (BIO4) was the second most important variable, contributing 17.7% and showing a permutation importance of 22.5%, followed by precipitation seasonality (BIO15), which had a percent contribution of 22.2% and permutation importance of 10.8% (Table 1).

In addition to the spatial shifts in distribution, a variation was observed in the extent of areas classified as suitable and highly suitable across climate scenarios. Under current climate conditions, the estimated area of suitable habitat (0.5–0.75) is 63,870 km^2^, while highly suitable habitat (0.75–1) covers 39,915 km^2^.

**FIGURE 3:**
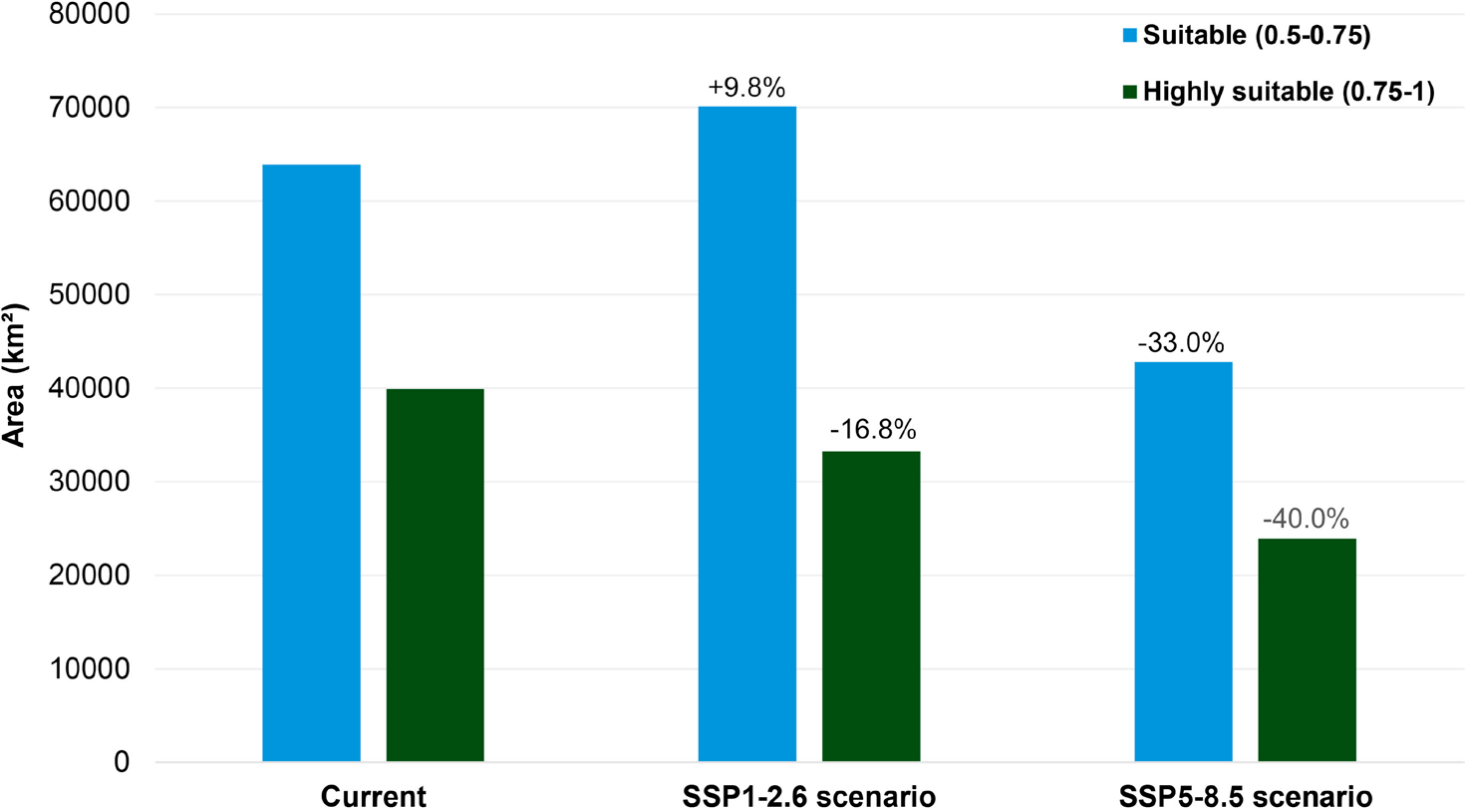
Projected changes in suitable (0.5–0.75) and highly suitable (0.75–1) habitat areas (km^2^) for *Hoplias malabaricus* (BOLD:AFU2064) under current, SSP1–2.6, and SSP5–8.5 climate scenarios. Percent variation relative to the present is indicated.

Under the SSP1–2.6 scenario (low greenhouse gas emissions), the extent of suitable habitat increases by approximately 9.8% to 70,147 km^2^, whereas the area of highly suitable habitat decreases by about 16.8% to 33,217 km^2^, with a possible shift in habitat quality, with a broader but potentially less optimal environmental envelope. In contrast, under the SSP5–8.5 scenario (high emissions), a pronounced decline is projected in both habitat categories: suitable habitat decreases by approximately 33.1% to 42,766 km^2^, and highly suitable habitat drops by 40% to 23,936 km^2^, with a trend toward habitat contraction and degradation, especially for optimal conditions, under more severe climate change projections (Figure 3).

**FIGURE 4:**
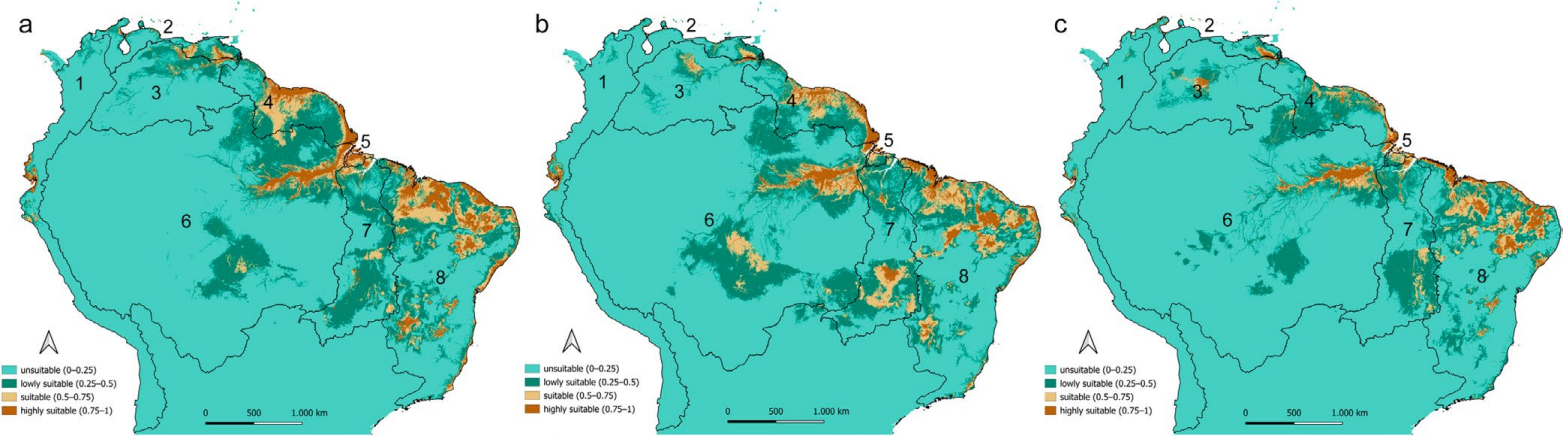
Geographic distribution of suitable habitats for *Hoplias malabaricus* (BOLD:AFU2064) under (A) current, (B) SSP1–2.6, and (C) SSP5–8.5 climate scenarios. Major hydrographic basins are indicated by numbers: (1) Pacific Coast, (2) Maracaibo–Caribbean, (3) Orinoco, (4) Guiana Shield, (5) Marajó Island, (6) Amazon, (7) Tocantins–Araguaia, and (8) Brazilian Atlantic Coast.

The spatial comparison of habitat suitability across the present and two future climate scenarios presents notable shifts in the distribution of environmentally favorable areas for the species. Under current conditions (Figure 4A), highly suitable habitats are primarily concentrated along the Guiana Shield, eastern Amazon and North portion of the Brazilian Atlantic Coast, forming relatively continuous and dense patches. In the SSP1–2.6 scenario (Figure 4B), these core areas are largely maintained, although some regions, particularly in the southern Tocantins‐Araguaia, show an expansion of suitable and highly suitable areas, suggesting potential habitat gains under moderate climatic change.

In contrast, the SSP5–8.5 scenario (Figure 4C) indicates more fragmented and spatially restructured patterns. While some key regions like the Guianas and parts of the eastern Amazon still retain high suitability, several areas exhibit a reduction in habitat continuity, with formerly suitable zones becoming dominated by low suitability or even unsuitable areas. Notably, suitability emerges in areas that are currently less favorable, reflecting a geographic displacement of optimal habitat conditions (e.g., Orinoco basin). While certain areas may persist as climatic refuges, the species' overall suitable habitat may undergo spatial fragmentation and reorganization, particularly under more severe emission trajectories.

### 3.3 Niche Across Climate Scenarios

Comparative analysis of the environmental ranges associated with suitable (0.5–0.75) and highly suitable (0.75–1) habitats across the current and future climate scenarios (SSP1–2.6 and SSP5–8.5) reveals distinct patterns of niche shifts for *H. malabaricus* . Elevation exhibited relatively consistent ranges across all scenarios, though a slight expansion in upper elevation limits was observed under SSP1–2.6 for suitable habitats (reaching up to 1114 m), compared to 907 m in the present and 880 m under SSP5–8.5. Highly suitable areas, however, showed slightly narrower elevation ranges in future projections.

Temperature seasonality (BIO4) displayed broad environmental tolerance across all scenarios, with little variation in maximum values for highly suitable habitats: 277.2% in the present, 250.7% under SSP1–2.6, and 280.6% under SSP5–8.5. Interestingly, the lower bounds decreased in future scenarios, indicating potential expansion into more climatically stable regions (14.4% in SSP1–2.6 compared to 26% in the present).

Precipitation seasonality (BIO15), one of the most influential variables in SSP5–8.5, showed substantial expansion in its range, especially under this high‐emission scenario. While highly suitable habitats tolerated values up to 159% in SSP1–2.6 and 156.2% in the present, this threshold increased to 207.6% in SSP5–8.5, suggesting greater resilience to rainfall variability under extreme conditions.

Regarding precipitation of the warmest quarter (BIO18) and coldest quarter (BIO19), broader ranges were observed under SSP1–2.6 compared to the present. For instance, BIO18 reached values up to 1393 mm (suitable) and 1132 mm (highly suitable) in SSP1–2.6, against 953 mm and 879 mm in the present. BIO19 showed a similar trend, with upper bounds extending to 1570 and 1447 mm, respectively, under SSP1–2.6. These variables were not included in the SSP5–8.5 scenario due to their lower relative importance, reinforcing the shift in ecological drivers under different climate futures.

Overall, the results indicate that while the species maintains environmental plasticity, particularly in relation to temperature and elevation, future climate scenarios may impose constraints on key hydrological variables, potentially reshaping the species' ecological niche.

The environmental ranges associated with the most important variables for each scenario are summarized in Table 2, while complete data for all variables included in the models can be found in File S1.

**TABLE 2:** Environmental range (min–max) of key variables for *Hoplias malabaricus* (BOLD:AFU2064) in suitable (0.5–0.75) and highly suitable (0.75–1) habitats under current, SSP1–2.6, and SSP5–8.5 scenarios.

Variable	Current		SSP1–2.6		SSP1–2.6	
	Suitable (0.5–0.75)	Highly suitable (0.75–1)	Suitable (0.5–0.75)	Highly suitable (0.75–1)	Suitable (0.5–0.75)	Highly suitable (0.75–1)
DEM	**−32 to 907**	**−35 to 800**	**−32 to 1114**	**−35 to 782**	**−32 to 880**	**−35 to 820**
BIO4	**26 to 280.8**	**30.4 to 277.2**	**14.4 to 279.2**	**19.6 to 250.7**	**28.8 to 296.9**	**30.2 to 280.6**
BIO15	10.5 to 198.1	11.4 to 198.1	**35.5 to 181.1**	**40 to 159**	**23.1 to 207.3**	**28.1 to 207.6**
BIO18	**3 to 953**	**10 to 879**	**0 to 1393**	**1 to 1132**	0 to 1076	0 to 858
BIO19	**0 to 1676**	**0 to 1641**	**0 to 1570**	**3 to 1447**	0 to 2114	0 to 2141

*Note:* Values in bold indicate variables of highest importance for each scenario.

### 3.4 Regional Patterns of Habitat Suitability

Initial analyses considering the entire distribution range of *H. malabaricus* revealed a wide amplitude in key environmental variables (e.g., elevation, temperature seasonality, precipitation seasonality). These patterns point to the potential existence of niche segregation mechanisms across the landscape. To explore this further, we conducted a spatially explicit analysis by hydrographic basin. This approach allowed for a more detailed examination of the environmental contexts associated with habitat suitability in each region and enabled comparisons under current and projected climate scenarios.

To ensure a more ecologically grounded interpretation of habitat suitability, basin‐level analyses were limited to regions with confirmed occurrence records of *H. malabaricus* (AFU2064), namely the Brazilian Atlantic Coast, Guianas Shield, Amazon, Marajó, and Tocantins‐Araguaia basins. Although the MaxEnt model also predicted potential suitable areas in other basins (e.g., Orinoco, Pacific Coast, Maracaibo‐Caribbean), these regions lack current occurrence data and were therefore included in File S2 only for exploratory purposes.

In the Amazon basin, suitable habitat is expected to increase by 75.4% under the SSP1–2.6 scenario (from 10,173 to 17,846 km^2^), while highly suitable areas remain relatively stable (+2.6%). However, under SSP5–8.5, both suitable and highly suitable areas are projected to decrease by 27.5% and 24.5%, respectively. For the Atlantic Coast of Brazil, both future scenarios predict a reduction in habitat extent. Suitable areas declined by 11.5% in SSP1–2.6 and 28.4% in SSP5–8.5, while highly suitable areas decreased by 27.3% and 39.2%, respectively, indicating a sharp contraction in optimal habitat conditions. In the Guiana Shield, the reduction is even more pronounced. Suitable habitat area drops by 17.5% in SSP1–2.6 and by 60.3% in SSP5–8.5. Highly suitable areas decrease by 10% and 63.8%, respectively. The Tocantins‐Araguaia basin shows a contrasting pattern, with suitable habitat more than doubling under SSP1–2.6 (+102.7%) and decreasing only slightly under SSP5–8.5 (−15.4%). Highly suitable areas increase by 82.3% in SSP1–2.6 but drop sharply (−50.4%) under the high‐emission scenario. Finally, in the Marajó Island basin, changes are less pronounced. Suitable habitat remains relatively stable, with a 10.7% decrease under SSP1–2.6 and a 14.3% increase under SSP5–8.5. However, highly suitable areas decreased by 85.2% in SSP1–2.6 and 75.4% in SSP5–8.5, indicating a substantial loss of optimal conditions despite stable suitability overall (Table 3).

**TABLE 3:** Projected changes (%) in suitable (0.5–0.75) and highly suitable (0.75–1) habitat areas for *Hoplias malabaricus* (BOLD: AFU2064) across major hydrographic basins under future climate scenarios (SSP1–2.6 and SSP5–8.5), compared to present conditions.

Hydrographic basin	Suitability class	Current (km^2^)	SSP1–2.6 (km^2^)	Change (%)	SSP5–8.5 (km^2^)	Change (%)
Amazon	Suitable (0.5–0.75)	10,173	17,846	+75.4%	7371	−27.6%
	Highly suitable (0.75–1)	7839	8043	+2.6%	5916	−24.5%
Brazilian Atlantic Coast	Suitable (0.5–0.75)	29,591	26,189	−11.5%	21,186	−28.4%
	Highly suitable (0.75–1)	19,685	14,314	−27.3%	11,976	−39.2%
Guiana Shield	Suitable (0.5–0.75)	11,277	9306	−17.5%	448	−60.3%
	Highly suitable (0.75–1)	6665	5999	−10.0%	241	−63.8%
Tocantins‐Araguaia	Suitable (0.5–0.75)	5694	11,544	+102.8%	4818	−15.4%
	Highly suitable (0.75–1)	1063	1937	+82.2%	527	−50.4%
Marajó Island	Suitable (0.5–0.75)	915	817	−10.7%	1046	+14.3%
	Highly suitable (0.75–1)	1017	151	−85.1%	250	−75.4%

*Note:* Positive percentages (green shades) indicate expansion area, while negative values (salmon shades) indicate area loss compared to the present scenario.

### 3.5 Niche Across Climate Scenarios

#### 3.5.1 Brazilian Atlantic Coast

In suitable habitats, elevation ranged from −24 to 907 m in the present, shifting slightly to −26 to 865 m under SSP1–2.6 and −26 to 880 m in SSP5–8.5. Temperature seasonality (BIO4) ranged from 29.2–242 in the present, increasing to 30.9–239.2 in SSP1–2.6 and further to 39.3–296.9 in SSP5–8.5. Precipitation seasonality (BIO15), not relevant in the present, ranged from 35.5–127.1 (SSP1–2.6) to 31.5–119.8 (SSP5–8.5). These shifts suggest a trend toward seasonal environments. In highly suitable habitats, elevation remained stable across scenarios (−32 to 800 m, −32 to 777 m, −32 to 820 m), while BIO4 increased from 30.4–233.6 to 28.4–230.5 (SSP1–2.6) and 39.8–276.2 (SSP5–8.5). BIO15 followed a similar trend (40–129.6 to 55.6–124.9), indicating a stronger association with seasonal climates over time.

#### 3.5.2 Guianas Shield

In suitable habitats, elevation was relatively narrow and stable (−22 to 450 m in the present, −28 to 483 m in SSP1–2.6, and −30 to 410 m in SSP5–8.5). However, BIO4 increased from 37.5–89.1 (present) to 48.7–110.2 (SSP1–2.6) and 78–200.5 (SSP5–8.5). BIO15 followed a similar trend: from 41.4–102.7 to 60.6–134.7, showing a shift toward highly seasonal climates. In highly suitable zones, elevation rose slightly −35 to 175 m (current), −35 to 283 m (SSP1–2.6), −35 to 403 m (SSP5–8.5), while BIO4 increased from 36.3–90.2 to 42–109.7 and 68.9–192.9. BIO15 also became more variable (47.5–95.2 to 69–133.6).

#### 3.5.3 Amazon

In suitable areas, elevation shifted from −18 to 467 m (present) to −22 to 485 m (SSP1–2.6), and −20 to 328 m (SSP5–8.5). Temperature seasonality (BIO4) rose from 26–145.1 to 52.8–156.2 and then to 105.3–281.1. Precipitation seasonality (BIO15) expanded from 41.3–103.2 to 52.1–128.9.

In highly suitable zones, elevation dropped considerably: from −25 to 175 m to −25 to 336 m and −25 to 157 m, indicating increasing preference for lower altitudes. BIO4 increased significantly 32.9–88.7 (current), 71.6–140.9 (SSP1–2.6), 105.3–280.6 (SSP5–8.5), and BIO15 rose from 43.3–87.8 to 62–123.1, showing marked seasonal preference.

#### 3.5.4 Marajó Island

Due to the region's geography, elevation was consistently low: suitable class ranged from −12 to 31 m (present), −21 to 29 m (SSP1–2.6), and −22 to 28 m (SSP5–8.5); highly suitable remained similarly constrained (−22 to 29, −22 to 28, −18 to 24).

Temperature seasonality (BIO4) increased across the board: 43.5–83.7 → 66.5–91.7 → 97.1–131.8 (suitable) and 43.6–84.7 → 75.3–92.5 → 85–133.1 (highly suitable). BIO15 showed a rise in variability from 56.9–84.2 to 71.9–94.1 and 75.5–97.9.

#### 3.5.5 Tocantins‐Araguaia

In suitable habitats, elevation slightly decreased: −32 to 788 m (present), −32 to 806 m (SSP1–2.6), −32 to 733 m (SSP5–8.5). Temperature seasonality (BIO4) rose substantially: 35.9–188.9 → 54.1–212.4 → 88.9–260.1. BIO15 expanded from 54.3–92.9 to 69.6–101.6, showing stronger seasonal preference. Highly suitable zones followed the same trend for the BIO4 increasing from 37.2–185.4 (current), 53.9–150.9 (SSP1–2.6), 89.1–206.4 (SSP5–8.5), and BIO15 from 62.5–92.2 (SSP1–2.6) to 73.3–95.2 (SSP5–8.5).

Overall, the results reveal a consistent trend of climatic suitability in more seasonal regions across future scenarios. While the specific ranges of environmental variables such as elevation, temperature seasonality (BIO4), and precipitation seasonality (BIO15) vary among basins and between suitability classes, it is evident that the areas remaining suitable and highly suitable for the species tend to be characterized by greater climatic variability. This is particularly noticeable in the increasing association with higher values of BIO4 and BIO15 in several basins under both SSP1–2.6 and SSP5–8.5 scenarios. These findings suggest that, under future climate conditions, the species may persist preferentially in regions with broader annual fluctuations in temperature and precipitation, indicating a shift in the spatial and climatic profile of its most favorable habitats.

## 4 Discussion

The ecological niche models generated for *H. malabaricus* exhibited strong performance across all climate scenarios, with AUC values consistently exceeding 0.9, classifying the models as excellent (Swets 1988; Phillips et al. 2006). This robust performance indicates a high level of agreement between model predictions and the species' known distribution, providing confidence in the projections under future climate conditions.

Among the environmental variables tested, temperature seasonality (BIO4) and elevation consistently emerged as the most influential across scenarios. Temperature affects fish metabolic performance and reproductive success (Beachum et al. 2020; Patel 2025), while variations in elevation shape river gradients, floodplain extent, and habitat accessibility (Fernando et al. 2025). This pattern suggests that *H. malabaricus* is particularly sensitive to altitudinal gradients and seasonal thermal fluctuations, which align with previous studies highlighting the role of these factors in shaping freshwater fish distributions (Schaefer and Arroyave 2010; Van Vliet et al. 2013). Other variables, such as precipitation of the warmest quarter (BIO18) and precipitation seasonality (BIO15), gained relative importance under future scenarios, suggesting a potential shift in the climatic constraints defining suitable habitats for the species. Although less influential in current models, precipitation‐related factors can affect flood‐pulse dynamics and nutrient inputs, which in turn sustain planktonic prey communities (Sánchez‐Hernández et al. 2021).

Overall, the species demonstrates a preference for low to mid‐elevation areas and a notable tolerance to temperature seasonality and hydrological extremes. However, under the optimistic SSP1–2.6 scenario, there is a trend toward occupancy of less seasonal environments, with the lower limit of BIO4 for suitable areas decreasing from 26% (present) to 14.4%. This shift suggests a potential movement toward a climatically more stable region. Precipitation seasonality (BIO15), which was not important in the current scenario, became influential in future projections, especially under SSP5–8.5. The broader range of this variable, reaching up to 207.6% in highly suitable areas, indicates an increased resilience to hydrological extremes, an adaptive trait that could be important given the projected rise in extreme events across the Amazon region and surroundings (Da Silva et al. 2019; Liang et al. 2020).

Precipitation‐related variables such as BIO18 and BIO19 (precipitation of the warmest and coldest quarters, respectively) showed increased tolerance ranges under SSP1–2.6. For example, the upper limit of BIO18 rose from 953 mm (present) to 1393 mm, suggesting greater hydrological flexibility under moderate conditions. In contrast, under SSP5–8.5, these variables were not among the most influential, potentially indicating a shift in ecological pressures.

In the most extreme scenario (SSP5–8.5), elevation remained the dominant variable, while temperature and precipitation seasonality played increasingly prominent roles. This likely reflects growing climatic instability under high‐emission scenarios, especially in tropical regions (Tretkoff 2011; Allan and Liu 2019). These findings underscore the multidimensional climatic niche of *H. malabaricus* , shaped by both thermal and hydrological gradients, and suggest some degree of resilience, at least within its core ecological preferences.

In the Atlantic Brazil basin, the species increasingly associates with more seasonal environments, as indicated by rising BIO15 values in highly suitable areas, peaking at 207.6% under SSP5–8.5. Despite high deforestation rates and limited protected area coverage (Klink and Machado 2005; Teixeira et al. 2021), this region hosts the largest projected extent of highly suitable habitat (19,685 km^2^), emphasizing its role as a potential climate refuge and the urgent need for targeted conservation efforts in unprotected areas. In the Guiana Shield, likely the species type locality and home to a genetically isolated population (Guimarães et al. 2022), highly suitable areas are projected to shift to slightly higher elevations (up to 410 m). Although this may reflect a response to worsening climatic conditions, it does not prevent a loss of 63.8% of highly suitable habitat under SSP5–8.5. The region's limited connection with other basins, coupled with its distinct genetic structure, underscores the need for focused conservation strategies. In the Amazon Basin, suitable habitats shift to lower elevations under the most severe scenario, down to 157 m. This trend may be related to changes in regional precipitation patterns; predictions suggest more intense droughts in the east and increased rainfall in the west (Sorribas et al. 2016). Most highly suitable habitats are concentrated along major rivers and floodplains, especially in the middle and lower Amazon. However, these areas are poorly covered by protected areas, increasing their vulnerability to both climate change and human pressures (Abell et al. 2017). As Frederico et al. (2021) noted, over 25% of threatened freshwater species are found in large river systems with insufficient protection, revealing a critical gap in conservation planning. Marajó Island, characterized by seasonal environments due to its unique geography, is projected to lose 75.4% of its highly suitable habitat under SSP5–8.5. This would also compromise its current role as an ecological corridor between the Atlantic, Guianas, and Amazon basins, increasing population isolation and reducing long‐term viability. In the Tocantins‐Araguaia basin, the species also shows increasing affinity for highly seasonal environments, in line with rising BIO15 values across scenarios. However, under SSP5–8.5, suitability drops sharply, with potentially viable areas restricted to the southern portion of the basin in the milder scenario. Whether the species can effectively reach and persist in these new areas will depend on the degree of hydrological connectivity, since *H. malabaricus* is a sedentary fish (Bialetzki et al. 2002).

Quantitative habitat suitability analyses revealed contrasting trends across climate change scenarios, highlighting the dynamic response of *H. malabaricus* to future environmental conditions. Under current conditions, the species benefits from a wide extent of suitable (63,870 km^2^) and highly suitable habitat (39,915 km^2^), reflecting its broad ecological tolerance. In SSP1–2.6, suitable habitat increases moderately (+9.8%), while highly suitable areas decline (−16.8%), suggesting a shift from optimal to marginal conditions. This trade‐off may allow for persistence but could alter population density and demographic structure across its range.

Under the high‐emission SSP5–8.5 scenario, both suitable and highly suitable areas experience substantial reductions (−33.1% and −40%, respectively), reflecting ecological degradation that may compromise population persistence. The sharp decline in ideal habitats raises concerns about the species' vulnerability under extreme climate change, especially if dispersal and ecological plasticity are insufficient to buffer against rapid environmental shifts (Moritz and Agudo 2013).

Our results reveal a predominant pattern of persistence through habitat contraction, rather than range expansion, under future climate change scenarios. This outcome is likely driven by two main factors: the broad distribution of the species and its low habitat specificity. Nevertheless, wide distribution and recognized environmental tolerance do not guarantee resilience to projected climate change. In fact, results point to significant losses in both suitable habitat and ecological connectivity, raising concerns about population fragmentation. Given the strong genetic structuring among *H. malabaricus* populations (Guimarães et al. 2022), such fragmentation could disrupt metapopulation processes and increase the risk of local extinctions. Many freshwater fish depend heavily on seasonal hydrological cycles (Smith et al. 2020; Sauz‐Sánchez et al. 2021), and for *H. malabaricus* , reproductive cycles are tied to seasonal changes and require shallow spawning grounds (Araujo‐Lima and Bittencourt 2001; Marques et al. 2001). Thus, while future climate conditions may still support areas of climatic suitability, actual persistence will depend on the continued availability of key hydrological and reproductive conditions.

Despite the robust performance of the ecological niche models, it is essential to acknowledge their inherent limitations and associated uncertainties. First, climate projections are subject to variability among GCMs and emission scenarios. Additionally, our models assume equilibrium between species distribution and environmental variables, without accounting for dispersal limitations, biotic interactions, or potential evolutionary adaptations. The reliance on current occurrence records may also introduce sampling biases, particularly in under‐surveyed regions. These limitations underscore the need for future validation efforts, combining long‐term monitoring, genetic data, and occurrence tracking under changing climatic conditions.

From a conservation standpoint, the projected loss of suitable habitats and reduction in ecological connectivity highlight the importance of incorporating climate adaptation into freshwater biodiversity planning. The identification of climatically stable areas can inform the design of climate refuges and ecological corridors. These corridors are critical for sustaining gene flow among fragmented populations, especially given the strong genetic structuring of *H. malabaricus* (Guimarães et al. 2022) and its limited dispersal ability. Future research should integrate habitat suitability models with landscape connectivity analyses and hydrological forecasting to better anticipate conservation bottlenecks and prioritize mitigation strategies.

In summary, although *H. malabaricus* displays considerable ecological plasticity, our findings suggest that climate change will challenge the species' resilience, particularly in isolated or marginal habitats. Ensuring long‐term persistence will require targeted conservation strategies that account for climatic projections and for the ecological and genetic distinctiveness of regional populations.

## Author Contributions

**Karen L. Auzier Guimarães:** conceptualization (equal), formal analysis (equal), methodology (equal), project administration (equal), validation (equal), writing – original draft (equal), writing – review and editing (equal). **Sarah J. do Nascimento Andrade:** methodology (equal), writing – review and editing (equal). **Luís R. Ribeiro Rodrigues:** conceptualization (equal), resources (equal), supervision (equal), validation (equal), writing – review and editing (equal).

## Conflicts of Interest

The authors declare no conflicts of interest.

## Supporting information


**File S1:** Full environmental ranges for all variables included in the models across scenarios.
**File S2:** Predicted suitable areas in different basins.

## Data Availability

All the required data is uploaded as Supporting Information.
